# Experimental Swap of *Anopheles gambiae*'s Assortative Mating Preferences Demonstrates Key Role of X-Chromosome Divergence Island in Incipient Sympatric Speciation

**DOI:** 10.1371/journal.pgen.1005141

**Published:** 2015-04-16

**Authors:** Fred Aboagye-Antwi, Nahla Alhafez, Gareth D. Weedall, Jessica Brothwood, Sharanjit Kandola, Doug Paton, Abrahamane Fofana, Lisa Olohan, Mauro Pazmiño Betancourth, Nkiru E. Ekechukwu, Rowida Baeshen, Sékou F. Traorè, Abdoulaye Diabate, Frédéric Tripet

**Affiliations:** 1 Centre for Applied Entomology and Parasitology, School of Life Sciences, Keele University, Staffordshire, United Kingdom; 2 Department of Animal Biology and Conservation Science, Faculty of Sciences, University of Ghana, Legon, Ghana; 3 Centre for Genomic Research, University of Liverpool, Liverpool, United Kingdom; 4 Malaria Research and Training Center, Faculty of Medicine and Dentistry, University of Mali, Bamako, Mali; 5 Institut de Recherche en Sciences de la Santé/Centre Muraz, Bobo-Dioulasso, Burkina Faso; University of Wisconsin–Madison, UNITED STATES

## Abstract

Although many theoretical models of sympatric speciation propose that genes responsible for assortative mating amongst incipient species should be associated with genomic regions protected from recombination, there are few data to support this theory. The malaria mosquito, *Anopheles gambiae*, is known for its sympatric cryptic species maintained by pre-mating reproductive isolation and its putative genomic islands of speciation, and is therefore an ideal model system for studying the genomic signature associated with incipient sympatric speciation. Here we selectively introgressed the island of divergence located in the pericentric region of the X chromosome of *An*. *gambiae* s.s. into its sister taxon *An*. *coluzzii* through 5 generations of backcrossing followed by two generations of crosses within the introgressed strains that resulted in *An*. *coluzzii*-like recombinant strains fixed for the M and S marker in the X chromosome island. The mating preference of recombinant strains was then tested by giving virgin recombinant individuals a choice of mates with X-islands matching and non-matching their own island type. We show through genetic analyses of transferred sperm that recombinant females consistently mated with matching island-type males thereby associating assortative mating genes with the X-island of divergence. Furthermore, full-genome sequencing confirmed that protein-coding differences between recombinant strains were limited to the experimentally swapped pericentromeric region. Finally, targeted-genome comparisons showed that a number of these unique differences were conserved in sympatric field populations, thereby revealing candidate speciation genes. The functional demonstration of a close association between speciation genes and the X-island of differentiation lends unprecedented support to island-of-speciation models of sympatric speciation facilitated by pericentric recombination suppression.

## Author Summary

*Anopheles gambiae* is the most important vector of malaria in Africa. This species is undergoing speciation and a number of subpopulations have been identified which can produce viable hybrid offspring but are reproductively isolated through assortative mating and ecological adaptation. This complex structure provides an ideal system for studying the unique genetic and behavioural processes required for speciation. *Anopheles gambiae*’s subpopulations differ genetically in limited regions of their genomes called islands of speciation. Theoretical studies predict that these islands, characterized by restricted genetic rearrangements, may protect genes of assortative mating between emerging species, and are fundamental to the speciation process. We set out to test this prediction by performing complex genetic crosses between the sister species *Anopheles coluzzii* and *Anopheles gambiae s*.*s*. and creating recombinant strains differing only at their X-chromosome island of speciation. We show through behavioural studies that recombinant females consistently mated with matching island-type males thereby associating assortative mating genes with the X-island of divergence. By sequencing the genetic code of the recombinant strains and natural populations, we could confirm these findings and identify candidate assortative mating genes. These findings suggest an important role of divergence islands for the genetic and behavioural processes associated with speciation.

## Introduction

Unravelling the genomic processes underlying sympatric speciation, the evolution of new species from a single ancestral species within the same geographical region, is fundamental to our understanding of biodiversity. At the core of this interest is the search for distinct genomic signatures that can help us understand what is thought to be a relatively narrow and unlikely set of genetic and ecological conditions facilitating the emergence and divergence of two gene pools from an originally panmictic population. For sympatric speciation with gene flow to occur, divergent selection acting on locally adapted genes has to overcome the homogenizing effects of migration and recombination [1,2]. Theoretical models of sympatric speciation have long recognised that this can only occur under a restricted set of conditions in which the genomic architecture often plays a major role [1,3,4]. Features of the genome such as chromosomal inversions and peri-centromeric regions that suppress recombination and link together genes of pre-mating isolation and ecological adaptation genes are predicted to facilitate sympatric speciation [1,5,6,7]. In addition, hemizygosity and lower recombination rates are thought to predispose sex chromosomes to the more rapid accumulation of genes of pre and post-mating isolation [8].

These general predictions have found empirical support from a limited number of studies designed to map genes involved in speciation [9,10] and/or to detect loci under divergent selection across genomes [11,12,13,14]. Currently this evidence concerns almost exclusively species already separated by both pre-zygotic and intrinsic post-zygotic reproductive isolation in which teasing out the genomic signature of the onset of speciation from the genomic processes that follow post-mating reproductive isolation constitutes a major challenge [1,3,4]. The *Anopheles gambiae* complex comprises the main vector species responsible for malaria transmission. It is also a species complex rich in cryptic taxa separated by various degrees of reproductive isolation [15,16] and could provide the ideal system for studying the genomic signature of pre-mating isolation independent of intrinsic isolation processes. The sibling species *An*. *coluzzii* and *An*. *gambiae s*.*s*. were until recently known as the 'M and S' molecular forms of *An*. *gambiae* in reference to diagnostic genetic differences in the ribosomal DNA regions [17]. These incipient species co-occur over large areas of West Africa and do not exhibit intrinsic post-mating barriers to reproduction [18,19]. Across much of their sympatric range their integrity is maintained by strong assortative mating [20,21] resulting in rare hybrids and low levels of genetic introgression between the two taxa [22,23]. However, in the Western coastal countries of Guinea Bissau and Senegal hybrids can occur locally at much higher frequencies [24], resulting in a large hybrid zone between the incipient sibling species and high levels of genetic introgression [22,23].

Divergence between these species is thought to be driven by larval adaptation to different types of bodies of water [25,26]. Transplant experiments have shown that *An*. *coluzzii* larvae survive better than those of *An*. *gambiae* s.s. in habitats that are more permanent and rich in aquatic predators while the reverse is true in predator-free temporary water bodies [25,27]. Adults of both species have similar feeding and resting habits and mate in swarms at dusk in villages. Swarm site segregation is thought to contribute to assortative mating [28], but the occurrence of mixed swarms at various frequencies [21,28] points towards additional conspecific recognition mechanisms, possibly based on flight tones [29,30].

Several groundbreaking studies have shown that sympatric speciation in these two incipient species probably involved the divergence of a few ‘islands of divergence' that possibly contain clusters of speciation genes and located in areas of low recombination [31,32]. These putative 'islands of speciation' include 3 pericentromeric islands of divergence located on the X, 2L and 3L chromosomes as well as smaller islands located in the vicinity of inversion breakpoints [31,32]. Perfect linkage disequilibrium between the X, 2L and 3L islands was found in samples from sympatric populations of *An*. *coluzzii* and *An*. *gambiae* s.s. from central West Africa [32]. This pattern suggested very low gene flow between the sibling species and the possibility that pericentromeric islands of divergence were merely 'incidental rather than instrumental' to the speciation process [32]. Subsequent genomic studies reinforced this view, suggesting divergence at many other loci across the genome and a more advanced stage of sympatric speciation [33]. However, recent studies have shown that the linkage disequilibrium between the pericentromeric islands breaks down to various degrees in areas with higher introgression between the sibling species [23,24]. Taken together the comparative genomics data would therefore support a model of genomic divergence in which pericentromeric divergence islands could play a major role in speciation in the face of varying levels of gene flow [22,23].

Since this view is currently subject to debate and because there are limits to the inferences that can be drawn from comparative genomics studies, we set out to demonstrate the role of divergence islands in the sympatric speciation process using an experimental functional genomics approach. We hypothesized that the largest putative 'speciation island' of the X chromosome would be a prime candidate for protecting assortative mating and ecological adaptation genes in the face of ongoing gene flow because it combines pericentromeric recombination suppression with the hemizygosity and decreased recombination typically associated with the X chromosome. Next, we selectively introgressed the S-form X-linked island of divergence of *An*. *gambiae* s.s. into an. *coluzzii* genetic background to create recombinant strains that shared an *An*. *coluzzii* genetic background but differed at their X-chromosome islands of speciation. Standardised assortative mating experiments were then used to test the potential association of the X-island molecular type with the mating preferences of recombinant and parental strains. These were combined with full-genome strain comparisons as well as targeted genome sequencing from sympatric populations. This strategy enabled us to identify the size of the pericentromeric region introgressed from *An*. *gambiae* s.s. into the *An*. *coluzzii* background, fixed protein-coding differences distinguishing recombinant strains, and conserved differences putatively relevant to speciation.

The results demonstrate the close association of assortative-mating genes with the X-island of speciation and thus lend support to models of speciation involving pericentric recombination suppression in these sympatric incipient species. In addition, the development of a laboratory-based model-system for studying assortative mating is an important step towards the description of key reproductive isolation genes and mechanisms responsible for pre-mating isolation among these cryptic taxa.

## Results

Recombinant strains with M or S-form X-island molecular type were produced by selectively introgressing the X-island from the S form Kisumu strain of *An*. *gambiae* s.s. into the M form Mopti strain of *An*. *coluzzii* for 4 generations, followed by two generations of crosses within the introgressed strain that resulted in *An*. *coluzzii*-like recombinant strains, one fixed for the M-type X-island (RbMM strain), and one with the S-type X-island (RbSS strain). The design ensured that, outside of the X-island region, the recombinant strains with an M-type X-island and that with an S-type one shared a large genetic similarity with the Mopti *An*. *coluzzii* strain. Additionally, recombinants strains shared the maternal mitochondrial genome from *An*. *gambiae* s.s. and the Y chromosome from *An*. *coluzzii*.

### Genetic characterisation of recombinant strains

Following their creation, the RbMM and RbSS recombinant strains and Mopti and Kisumu parental strains were further characterized by genotyping of their 2L and 3L pericentromeric divergence islands [32] and karyotyping of inversion polymorphisms on their 2L and 2R chromosome (Table 1). Predictably, the M-form Mopti strain exhibited M-type 2L and 3L islands in addition to its M-type X-island. It was also fixed for inversion *a* on chromosome 2L and polymorphic for *u* on 2R. Unexpectedly, the long-established S-form Kisumu strain was polymorphic at the 2L and 3L islands suggesting historical contamination with an M-form strain. The Kisumu was also polymorphic for *a* on 2L and standard on 2R. Amongst the recombinant strains, RbMM was M-like across all 3 islands. The RbSS was polymorphic at the 2L locus but fixed for the M-type allele at the 3L. Both recombinant strains were also polymorphic for *a* on 2L and *u* on the 2R chromosome (Table 1). Thus their genotypes and karyotypes were those expected from successful backcrossing and crossin steps.

**Table 1:** Recombinant and parental strains genotypes at the X, 2L and 3L divergence islands, and 2L and 2R inversion karyotypes.

Strain	Genotypic frequencies				Inversion frequencies		
	*N*	X	2L	3L	*N*	2L	2R
M Mopti	14	MM	MM	MM	20	*a*/*a*	+/*u*
S Kisumu	14	SS	0.2MM, 0.4MS, 0.4SS	0.6MM, 0.4MS	20	+/*a*	+
RbMM	20	MM	MM	MM	20	0.15*a*/*a*, 0.85+/*a*	0.25+/+, 0.75+/*u*
RbSS	21	SS	0.9MM, 0.1MS	MM	10	0.2*a*/*a*, 0.8+/*a*	0.4+/+, 0.6+/*u*

Genotypic and inversion frequencies and sample sizes are shown for the M Mopti and S Kisumu parental strains as well as the RbMM and RbSS recombinant strains.

### Behavioural assays of assortative mating in recombinants strains

**Fig 1:**
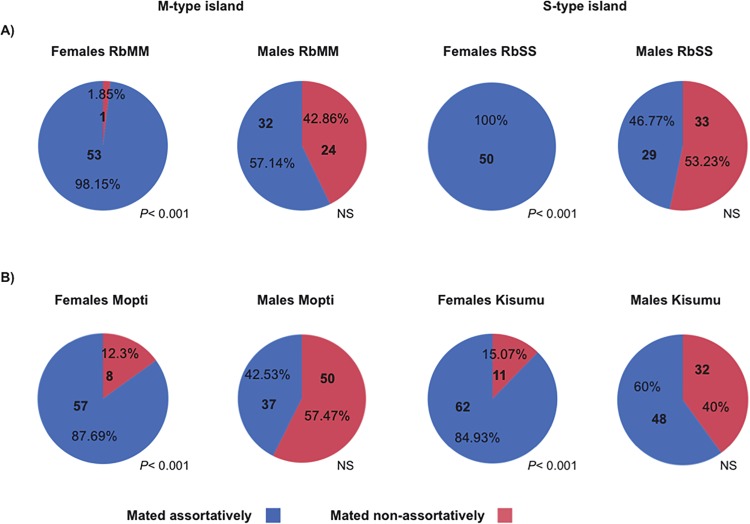
Percentage assortative mating in females and males carrying M or S-type X-chromosome islands. A) virgin females or males from the RbMM and RbSS recombinant strains where presented with a mixture of recombinant individuals of the opposite sex and with X-islands matching and non-matching their own X-island molecular type; B) virgin females or males from the M-form Mopti and S-form Kisumu strains used for creating the recombinant lines were given a choice between equal numbers of potential mates from both strains. The number and percentage of assortative and disassortative mating recorded across 3 replicates are indicated.

Next, the mating choice preferences of females and males were tested using a standardized assortative mating assay (see methods). Reciprocal experiments were conducted in which virgin females or males from the same RbMM and RbSS cohorts were given a choice between mates with matching or non-matching X-island type (Table 2). Females from the RbMM were found to mate almost exclusively assortatively (Fig 1A)(*P*< 0.001). Females RbSS had their mating preferences effectively swapped and mated entirely with recombinant males with matching S-type islands (Fig 1A)(*P*< 0.001). In contrast, males from the RbMM and RbSS recombinant strains did not significantly prefer females with matching X-islands (*P* = 0.284 and 0.611 respectively). Since assortative mating amongst laboratory strains from *An*. *coluzzii* and *An*. *gambiae* s.s has never been reported, female and male choosiness were also assessed in the Mopti and Kisumu parental strains. Here again, females mated significantly assortatively (*P*< 0.001 in both strains) but not males (*P* = 0.073 and 0.163)(Fig 1B and S1 Table).

**Table 2:** Number of females and males mating assortatively in reciprocal behavioural assays among the RbMM and RbSS recombinants strains.

**Replicate**	**Mating combination**				**Mating Type (%)**		**Chi-square**	***P*-value**
	**Females** ^**†**^		**Males** ^**†**^		**Assortative**	**Disassortative**		
1	MM		M	S	29	1	32.8	< 0.001
2	MM		M	S	24	0	33.3	< 0.001
*Both*					*53 (98*.*15)*	*1 (1*.*85)*	*64*.*9*	*< 0*.*001*
1	SS		M	S	25	0	34.6	< 0.001
2	SS		M	S	25	0	34.6	< 0.001
*Both*					*50 (100)*	*0 (0)*	*69*.*3*	*< 0*.*001*
**Replicate**	**Mating combination**				**Mating Type (%)**		**Chi-square**	***P*-value**
	**Males** ^**†**^	**Females** ^**†**^			**Assortative**	**Disassortative**		
1	M	MM		SS	15	16	0.1	0.857
2	M	MM		SS	17	8	3.3	0.069
*Both*					*32 (57*.*14)*	*24 (42*.*86)*	*1*.*1*	*0*.*284*
1	S	MM		SS	18	13	0.8	0.368
2	S	MM		SS	11	20	2.7	0.103
*Both*					*29 (46*.*77)*	*33 (53*.*23)*	*0*.*26*	*0*.*611*

Recombinant females (X-island genotypes MM or SS) were given a choice between recombinant males with X-chromosome speciation island matching their own or not (top half of table, see methods). The reciprocal experiments were also conducted with recombinant males (X-island genotype M or S) choosing recombinant females (bottom half of table). The number of replicates, mating combinations, numbers and percentages (in brackets) of mating, and level of significance (Chi-square Likelihood-ratios) are indicated.

^**†**^ For each mating combinations, 2 replicates were conducted using 5-day-old mosquitoes reared from independent mosquito cohorts.

### Full-genome sequencing of the RbMM, RbSS and Mopti strains

**Fig 2:**
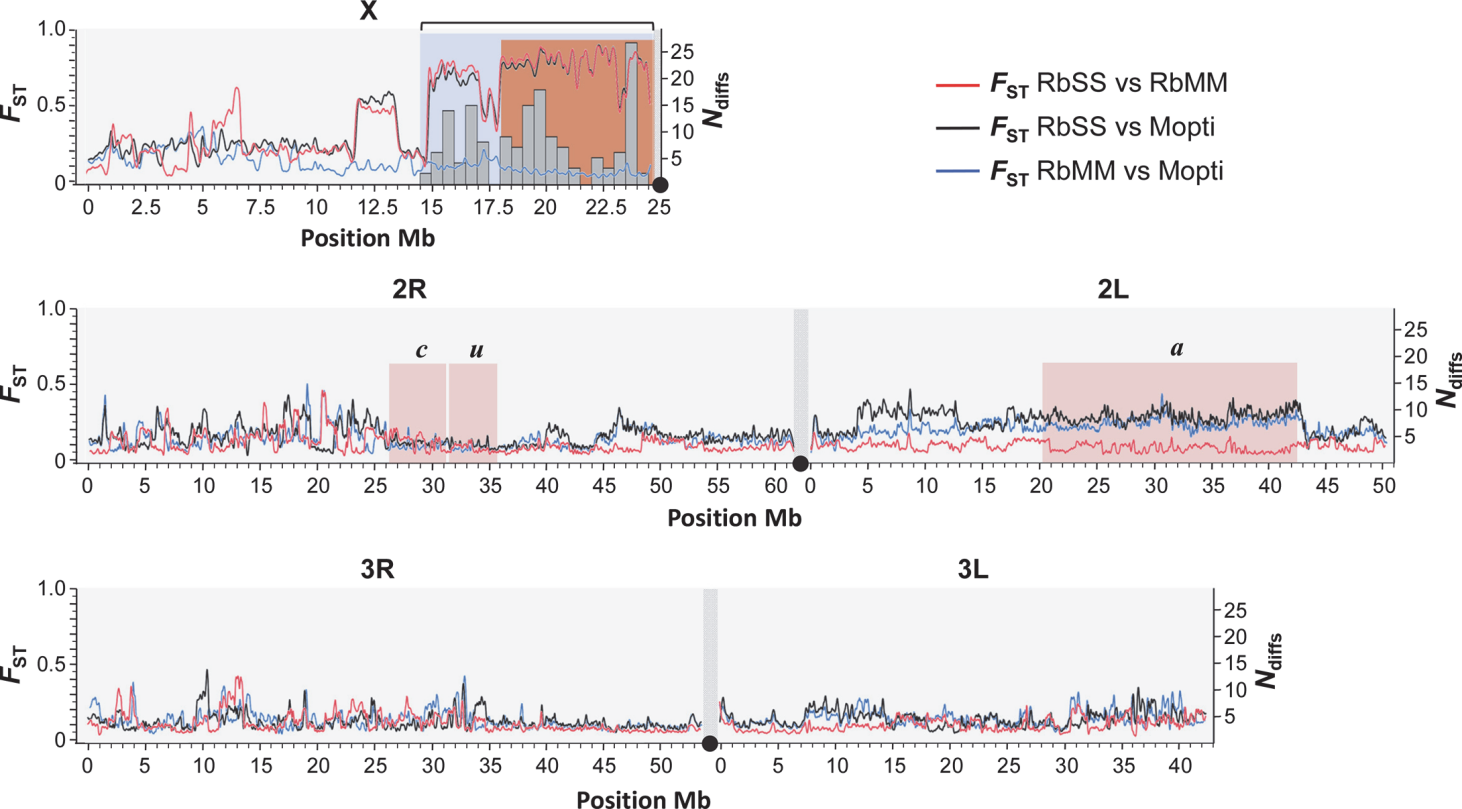
Genomic structure of recombinant strains. The genomes of the assortatively-mating RbMM, RbSS and parental Mopti strains were compared using *F*_ST_ estimates at ~3x10^6^ SNP marker loci (left Y-axis and red, blue and black lines). The genomic region introgressed from Kisumu into the Mopti genetic background and differing between the RbMM and RbSS recombinant strains is characterized by high *F*_ST_ values (blue shade) and extends from position ~14.5Mb to the centromere on chromosome X. The RbMM and RbSS differed at 160 protein-changing positions all of which located within the introgressed island and flanking region (right Y-axis, grey histogram bars). The pericentromeric region sharing conserved fixed differences with the field *Anopheles coluzzii and gambiae s*.*s* populations starts at position ~18.1Mb (orange shade). The position of inversions *c*, *u* and *a* on chromosome 2 is indicated (pink shade).

The size of the X-island of divergence and flanking regions differing between the RbMM and RbSS strains was determined through genome-wide genetic differentiation (*F*_ST_) scans and the occurrence of fixed coding differences between the recombinant strains. Estimates of genetic differentiation between the RbMM, RbSS and Mopti strains were calculated for 3,743,318 SNPs across the X, 2^nd^ and 3^rd^ chromosomes. The genome-wide *F*_ST_ scans showed that the RbSS differs from the RbMM and Mopti strains on chromosome X from the centromere to the reference position ~14.8Mb (Fig 2). This region covered the entire island of speciation plus a large flanking region. In addition, the RbSS and RbMM strain were genetically differentiated at a ~2Mb S-form fragment extending roughly from positions 11.5–13.5Mb (Fig 2). There were no other sizeable S-like regions detected through comparisons of the RbMM, RbSS and Mopti genomes, indicating that the selective introgression design worked as hoped for. Importantly, amongst all fixed differences observed between the RbMM and RbSS strains, non-synonymous differences inducing protein-coding changes (*n* = 160) were found only in the selectively-introgressed pericentromeric region (Fig 2). Fixed differences located elsewhere in the genome were either coding synonymous changes or non-coding.

### Comparison with X-island from sympatric field populations

**Fig 3:**
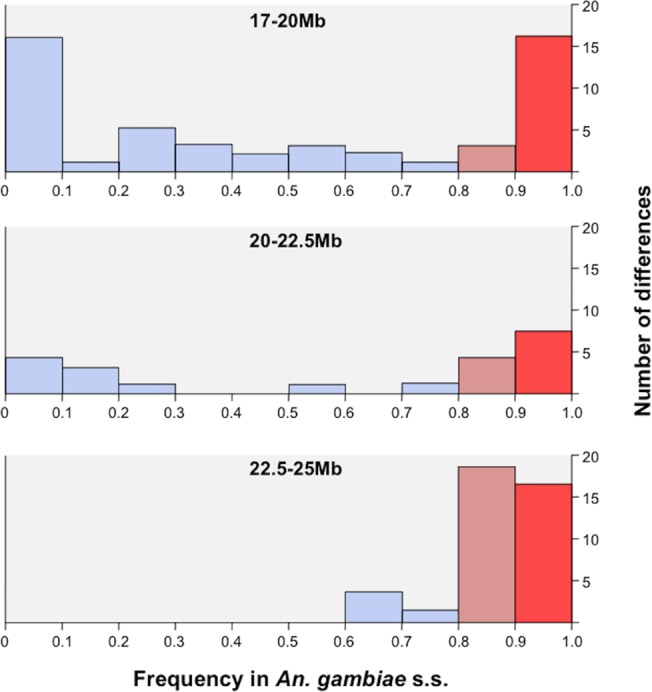
Field population frequency distribution of protein coding SNPs identified in RbSS. A region covering the X-island and flanking region up to reference position 17Mbp was captured and re-sequenced in sympatric *An*. *gambiae* s.s. and *An*. *coluzzii* populations from Ghana. The frequency of alleles coding for unique protein differences in the RbSS recombinant strain was measured in the field *An*. *gambiae* s.s. population. The proportion of alleles occurring at high 0.8 (orange bars) and very high (freq >0.95: red bars) frequency increased towards the centromere suggesting a potential role in speciation whilst other alleles (blue bars) were not conserved (see text for details).

Given that the Kisumu and Mopti strains were colonized from allopatric populations over 25 and 7 years ago, some of the differences observed between the RbMM and RbSS strains could be due to genetic divergence of the original populations or result from genetic drift and inbreeding [34]. Consequently, we compared the protein-coding differences identified between RbMM and RbSS with those observed between 2 sympatric *An*. *gambiae* s.s. and *An*. *coluzzii* populations from Southern Ghana. Deep-pooled-targeted exon re-sequencing of the region extending from 17Mb to the centromere showed that, in 114 of the 117 coding differences distinguishing RbSS from RbMM, the M-form allele was fixed or nearly fixed (freq >0.95) in the field population of *An*. *coluzzii*. In the sympatric *An*. *gambiae* s.s., the alternate S-type allele was found at frequencies >0.8 in 61 of the 114 differences; and 20 of those were fixed or nearly fixed (freq >0.95) and thus conserved differences between sibling species. Conserved differences started from position ~18.1Mb (Fig 2), increased in frequency with proximity to the centromere and affected a total of 12 genes (Fig 3, Table 3).

**Table 3:** Protein coding changes identified between the RbMM and RbSS strains confirmed in sympatric field populations of *An*. *coluzzii* and *gambiae* s.s.

Reference position	M-form allele	S-form allele	Codon (protein change)	Gene (putative function)
18314527	G	A	gGg/gAg (G74E)	AGAP013136
19052774	T	G	aAc/aCc (N25T)	AGAP001002 (Toll protein)
19114172	C	G	gGg/gCg (G232A)	AGAP001009
19114460	A	C	Tgg/Ggg (W160G)	AGAP001009
19114646	G	C	Ctg/Gtg (L98V)	AGAP001009
19116606	T	C	cAc/cGc (H197R)	AGAP013526
19636043	A	G	aAg/aGg (K272R)	AGAP001022 (gastrin/cholecystokinin receptor)
19636265	C	T	gCc/gTc (A346V)	AGAP001022
19637489	T	C	Tcg/Ccg (S355P)	AGAP001022
19714995	C	T	cGc/cAc (R491H)	AGAP001025 (protein msta)
19815325	A	T	Acg/Tcg (T339S)	AGAP001033 (mab-21 like protein)
20955148	G	A	aCg/aTg (T281M)	AGAP001050 (chondroitin polymerizing factor)
21093789	A	C	Ttt/Gtt (F288V)	AGAP001052 (ubiquitin carboxyl-terminal hydrolase)
22104989	T	C	Aat/Gat (N1296D)	AGAP001061
22159522	T	C	Atg/Gtg (M514V)	AGAP001061
22751398	A	G	Act/Gct (T252A)	AGAP001073
23799338	T	A	gaA/gaT (E879D)	AGAP013341
23799431	T	G	gaA/gaC (E848D)	AGAP013341
23799525	T	G	aAt/aCt (N817T)	AGAP013341
23799541	T	G	Att/Ctt (I812L)	AGAP013341

Twenty differences identified between the RbMM and RbSS recombinant strains were also fixed or nearly-fixed (freq >0.95) in sympatric field populations. These were distributed over 12 genes located within the ~ 6MB pericentromeric island of speciation.

## Discussion

This is the first functional genomic study aiming to test the role of the putative X-chromosome island of speciation in pre-mating reproductive isolation between *An*. *coluzzii* and *gambiae* s.s. and demonstrating its potential importance in their process of incipient sympatric speciation. Albeit demonstrated here using laboratory recombinant strains, the close association observed between pre-mating isolation genes and the X-island supports the hypothesis that pericentric regions can create linkage disequilibrium (LD) and thus protect associations between genes of pre-mating isolation and ecological adaptation [7] and facilitate the onset of sympatric speciation [1,6]. The results are consistent with the notion that hemizygosity and the lower recombination rates of sex chromosomes predispose them to accumulating genes of pre and post-mating isolation [8]. In the case of the X-island of speciation, the pericentric and X-chromosome recombination suppression effects synergise, thereby further reducing recombination and promoting high LD.

Although previous studies have linked loci responsible for reproductive isolation to inversions [12,13], pericentromeric regions [35,36] and sex chromosomes [9,10,35], this is one of the few studies focusing on incipient sympatric species in which intrinsic post-reproductive barriers to reproduction are not yet established and therefore investigating the genomic architecture of pre-mating isolation independent of intrinsic post-mating processes. In sulfur butterflies *Colias eurytheme* and *C*. *philodice*, pre-mating isolation was associated with an inversion located on the X-chromosome [37], but these species co-occur only over a limited hybridization zone. In the hawthorn and apple-infesting races of *Rhagoletis pomonella* flies genomic divergence was found accentuated around 'continents of differentiation' containing inversions on autosomes [38]. However, pre-mating isolation in this model system is ecological and incidental to specialization to different host plants, hence patterns of genetic divergence, migration and gene flow are more akin to those of micro-allopatric speciation.

Paradoxically, elucidating the processes underpinning the genomic structure of speciation of *An*. *coluzzii* and *An*. *gambiae* s.s. whose sympatric range spans large areas of Western Africa has proved challenging because of the contrasted patterns of introgression and selection observed within that range [17,22,23]. The experimental functional genomics approach taken here clarifies the role of so-called speciation islands in the speciation process. The results suggest a simple genetic mechanism whereby the low-recombining pericentromeric X-island enables these incipient species to maintain their genetic integrity in Central and Eastern areas of Africa where introgression is uncommon or temporally limited [20,23] and in the hybrid zones of coastal Western Africa where gene flow is extensive [23,24]. The results are compatible with model of sympatric speciation in which the X-chromosome island played an active role in speciation that involved 'divergence hitchhiking' around key speciation loci [1,3,4]. Whether the 2L and 3L islands have played similar important roles remains to be demonstrated. In this study we found no evidence of a direct association of the 2L and 3L islands with asssortative mating since the recombinant where either homozygous or nearly homozygous M-type for these regions. Therefore it is unlikely that these islands of differentiation play a major role in conspecific mate recognition.

In terms of physical size and the number of genes it contain, the X-island of speciation was the largest of the three pericentromeric islands described between *An*. *coluzzii and An*. *gambiae* s.s. through genome-wide studies [31,39]. Depending on the markers and populations considered, previous studies reported it as spanning 3–5Mb and as many as 75–200 genes [39,40]. Two studies showed that recombination was reduced by as much as 16 and 35-fold near the centromere compared to elsewhere on the X chromosome [40,41]. Based on our comparisons of sympatric populations from Ghana and the recombinant strains, we estimated that the island is over 6Mb-long and extends from positions ~18.1 to 24.2Mb. Within the island, 20 unique protein-coding changes affecting 12 genes were identified between the two sibling species. Amongst these genes, 6 have putative biological functions: *AGAP001002* (Toll protein) is involved in development and immunity and *AGAP001033* (mab-21 like protein) in neural and sensory organ development; *AGAP001050* (chondroitin polymerizing factor) and *AGAP001052* (ubiquitin carboxyl-terminal hydrolase) are involved in protein secretion and proteolysis; *AGAP001022* (gastrin/cholecystokinin receptor) is a receptor for peptides in the brain and gastrointestinal tract; and *AGAP001025* (protein msta) is involved in negative regulation of gene expression. Some of these genes might directly be involved with mating or interact with mating genes located elsewhere in the genome. However, others could also affect genes contributing to the ecological speciation of the sibling species, such as those responsible for form-specific larval habitat use [26,42] and larval predator avoidance behaviour [25,43].

Although we did not target intergenic regions when re-sequencing the X-island, these would warrant further investigation as they might contain regulatory elements with cis and trans effects on genes within the X, 2L and 3L island and possibly elsewhere in the genome. Trans-acting effects could explain the generally poor correspondence between differentially-expressed genes and islands of divergence observed in some *An*. *coluzzii* and *An*. *gambiae* s.s. populations [44,45]. In this study, the parental Mopti and Kisumu strains did not quite mate as perfectly assortatively as the RbMM and RbSS strains with homogenized genome. This would suggest that, in the parental strains, genetic interactions between the X-island and other parts of the parental genomes might have been responsible for a more variable mating phenotype. It should be note that although fixed non-synonymous coding differences were only identified within the selectively-introgressed X-island region, a number of non-coding differences were identified elsewhere in the genome. These might either be chance fixations due to the introgression process or be genuine differences between the parental Kisumu and Mopti strains. Therefore, and albeit we consider it highly unlikely, we cannot strictly rule out the possibility that a non-coding difference may have accidentally resulted in an assortative mating mechanism unique to the Kisumu and Mopti laboratory pairing and the resulting recombinant strains model system.

Despite the methodological complexities inherent in the creation of assortatively-mating recombinant strains and their behavioural and genomic characterization, the resulting laboratory-based model holds much promise for further characterization of assortative mating in *An*. *gambiae*. The occurrence of strict female mate-choice in behavioural assays implies the perfect phenotypic expression of species-specific recognition mechanisms in females as well as perfect cues in males. Although no male-driven mate choice was detected in the laboratory, this does not mean that males do not contribute to assortative mating in nature. In the field, males are known to contribute to assortative mating via swarm spatial segregation [21,28]. Furthermore, in large outdoor enclosure mate-choice experiments in which either virgin females or males of *An*. *coluzzii* were presented with an equal number of conspecific and interspecific mates, both females and males were found to mate significantly assortatively [46].

We are currently conducting finer phenotypic characterization of the RbMM and RbSS strains in an attempt to identify con-specific mate recognition mechanisms that result in assortative mating. Importantly, the availability of the RbMM and RbSS strains and the development of a standardized laboratory-based mating assay offers the possibility of direct validation of candidate reproductive isolation genes via knockdown and/or knockout experiments combined with accurate phenotyping. Unravelling the genetic basis of mate choice and assortative mating is not only relevant to our understanding of processes of speciation, it can also play a crucial role in improving the mating behaviour of anopheline strains that are mass-reared for sterile-male release programmes and the control of malaria.

## Materials and Methods

### Mosquito strains and rearing

The Kisumu strain of an *An*. *gambiae* s.s. (S molecular form) was used for selective introgression of its X-island of speciation into the Mopti strain of *An*. *coluzzii* (M molecular form) genetic background. The Kisumu strain was colonised over 25-years ago from the area of Kisumu, Kenya where *An*. *gambiae* s.s. populations are from the Savanna chromosomal form characterized by the presence of the *b* inversion on chromosome 2R [15,47,48]. The Mopti strain was colonized in 2003 by the Lanzaro lab (UC Davis) from the village of N’Gabacoro droit near Bamako, Mali, where *An*. *coluzzii* populations are characterized by the *bc* and *u* inversion polymorphisms on 2R typical of the Mopti chromosomal form [15,47,48]. Both strains are well adapted to the laboratory and lay eggs reliably, which was the most important consideration given the complexity of the envisaged genetic crosses.

The two strains were kept at 25°C±1°C and 70–80% relative humidity and reared under standard conditions in order to achieve homogeneity in phenotypic quality [49]. Adult females were fed on horse blood using an artificial feeder (Hemotek membrane feeding system, Discovery workshops, UK) and newly emerged first instars were reared in plastic trays (34x24cm) at a density of 200 larvae per tray in 1L of water. They were fed daily on ground fish food (Tetra werk, Melle, Germany). Pupae were placed in standard 5L rearing cages for emergence and newly emerged male and female mosquitoes were kept together and with access to 5% glucose solution at all time.

### Selective introgression of speciation islands

The genetic differences distinguishing the ribosomal DNA from the M molecular form *An*. *coluzzii* and S molecular form *An*. *gambiae* s.s. were originally described within the large X-chromosome island at a locus very near the centromere [50,51]. Using this marker, the island of speciation from the S molecular form Kisumu strain was introgressed into the M molecular Mopti strain background through 4 generations of selective introgression. The parental strains were first checked for possible contaminations by genotyping of the diagnostic rDNA IGS locus located in the X-chromosome island of speciation using the PCR-RFLP method developed by Fanello et al.[52]. Here and elsewhere 'M' and 'S' refer to the male genotype at the marker rDNA locus (males have one copy of the X-chromosome) and 'MM', 'SS' and 'MS' to possible homozygous and heterozygous female genotypes (females have two copies of the X-chromosome) at the same locus.

**Fig 4:**
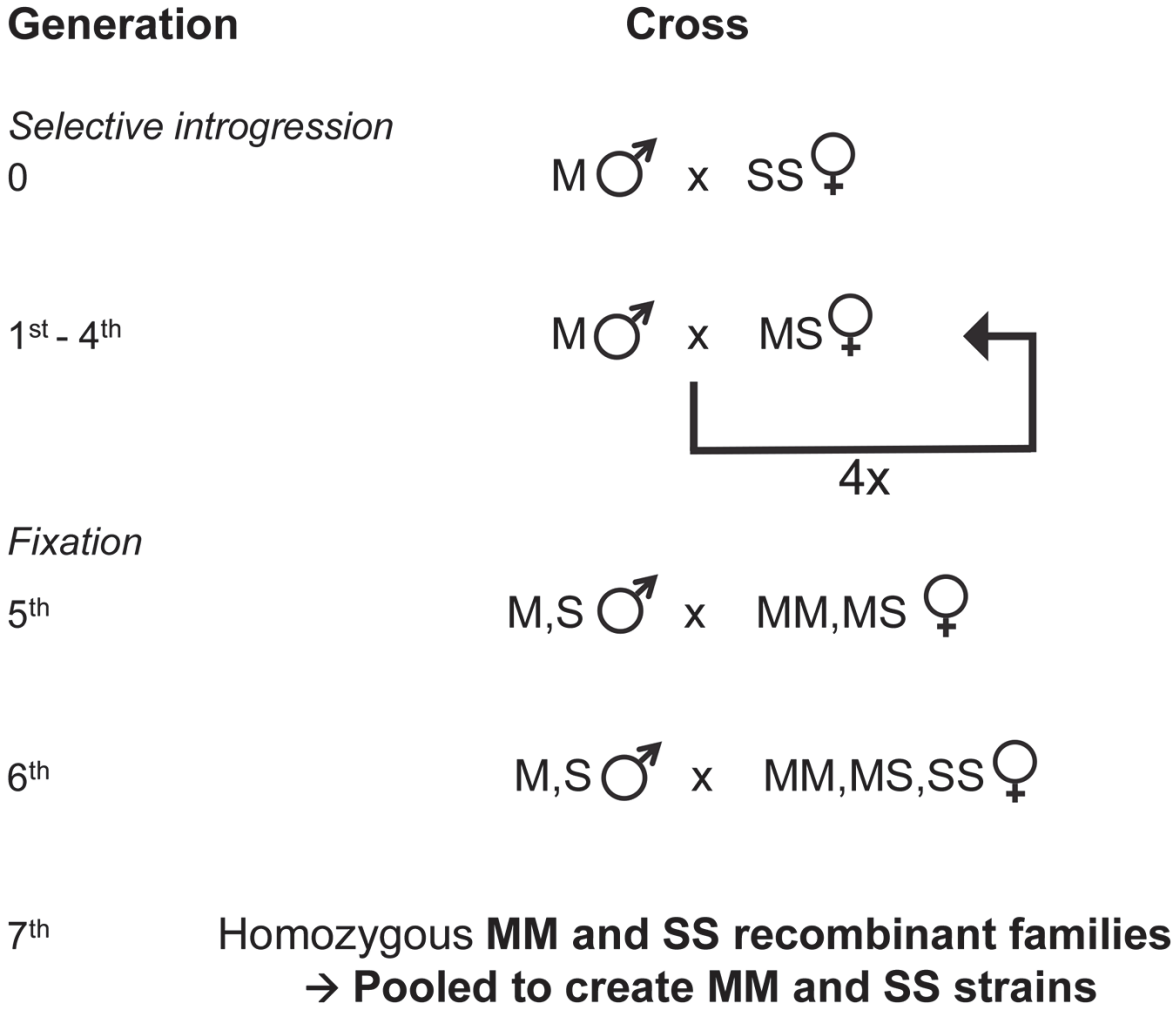
Genetic crossing design used for selective introgression of the X-island of divergence in recombinant strains. Following the creation of hybrid females at the X-island diagnostic rDNA locus, 4 generations of backcrosses were used to introgress the S-type X-island into an M molecular form Mopti genetic background. Here, 'MM', 'MS' and 'SS' refer to the female genotypes at the rDNA marker locus in the X-island and 'M' and 'S' refers to the male genotype at the same locus (see methods). Thereafter 2 generations of crosses within the introgressed strain resulted in recombinants strains that shared a high genetic identity but differed at the S or M-type X-chromosome islands of divergence.

Hybrids between the two strains were created by crossing 100 M Mopti males with 100 SS Kisumu females (Fig 4). In order to obtain the 1^st^ backcross progeny, 100 virgin MS hybrid females were mated with 100 virgin M Mopti males, resulting in male progeny of genotype M or S at the r-DNA locus and female of genotypes MM or MS (Fig 4). From generation 2 to 4, MS progeny females were backcrossed with M Mopti virgin males resulting in 4 generations of backcrossing (Fig 4). At each generation MS hybrid and MM families were obtained by bloodfeeding and setting up 80 females for individual oviposition. MS hybrid families were then distinguished from MM families by genotyping ten 2^nd^ instar larvae reared in individual trays. The progenies of trays identified as containing MS hybrid larvae was pooled and reared under standard rearing conditions to obtain the next generation MS backcross females. Families identified as MM were discarded.

### Creation of M and S X-chromosome islands recombinant strains

In order to obtain two strains with M and S X-island types but with high genetic similarity elsewhere in the genome, 2 generations of crosses within the introgressed strains were conducted. MS and MM females and M and S males from the 4^th^ backcross were randomly mated with one another in a mixed cage (100 males and 100 females) resulting in MM or MS 6^th^ generation families (Fig 4). Those progenies of families identified as being mixed M and S by larval genotyping which featured all possible male and female genotypes were randomly mated to one another in a mixed cage (100 males and 100 females) for a 7^th^ cross in order to generate MM, MS and SS families. The resulting MM and SS families were pooled together in order to obtain a pair of MM and SS strains, sharing a very large proportion of the Mopti genetic background but differing at their rDNA locus and linked X-chromosome island of speciation. The whole process was done twice simultaneously to generate two MM and two SS recombinant strains. Of those four strains, two were found to be heterozygous at the rDNA locus during their behavioural characterization suggesting possible contaminations. Therefore all analyses focus on the remaining MM and SS recombinant strains referred to as RbMM and RbSS throughout the text.

### Fitness of hybrid MS versus homozygous MM backcrosses

Following the first backcross generation, the ratio of MS to MM females (36/3 = 92.8%) significantly deviated from the expected 1:1 ratio (Chi-square Likelihood ratio: χ^2^ = 39.2, *df* = 1, *P*< 0.001) suggesting a strong hybrid advantage (S1 Fig). Thereafter no differences in the ratio of MS and MM females were found, with the percentage of MS females fluctuating from 46.2 in the 2nd (*P* = 0.579), to 64.8 in the 3rd (*P* = 0.0683), and 51.9 (*P* = 0.782) in the 4th backcross generations (S1 Fig). The proportion of MS to MM females in the fifth generation (1st cross within introgressed strain) did not significantly differ from the expected 3:1 MS/MM ratio (Chi-square Likelihood ratio: χ ^2^ = 0.19, *df* = 1, *P* = 0.664).

### Genotyping of the 2L and 3L speciation islands and identification of karyotypes

Following the publication of evidence showing linkage disequilibrium between the X, 2L and 3L islands in field M and S molecular form populations from West-Africa [32], the parental strains and recombinant strains were also characterized as ‘M-like or S-like’ at the 2L and 3L islands of speciation using archived DNA and the PCR-RFLP diagnostics developed by the same authors [32]. Primers and PCR conditions were as described by the authors except for the use of more sensitive AmpliTaq Gold DNA Polymerase (Life Technologies). Polytene chromosome preparations were made from the ovaries of 10–20 semi-gravid females per strain using established protocols [53,54]. Inversions present on chromosomes 2L and 2R were scored from chromosome spread under the light microscope.

### Behavioral assays of assortative mating

An assortative mating assay was developed in order to create conditions in which the mating choice preferences of females could be tested within standard laboratory breeding cages. Virgin males and females from each recombinant strain (RbMM and RbSS) were produced using our standardized rearing procedure (see first section) and kept in separate cages with access to 5% glucose solution at all times. In preliminary assortative mating experiments, 2-5-day-old virgin females from a given recombinant strain and X-chromosome island type were given a choice of 2-5-day-old recombinant males matching and non-matching their own X-island type. For each of 4 replicates, 30 females, 30 M-type and 30 S-type island males were placed in a standard 5L rearing cages with access to 5% glucose solution and given 24 hours to mate (6pm start). Infrared video recordings show that under these conditions, males initiated swarms soon after the insectary lights went off. At the end of these preliminary experiments all females were collected and stored in 70% ethanol until dissected for sperm genotyping. Across both mating combinations, the genetic analyses of the sperm extracted from the spermathecae of mated females showed that females mated preferentially with recombinant males with an X-island matching their own (S2 Table). Recombinant females with M-type X-islands mated with matching M-type X-island males on average 77% of the time (Chi-square = 15.9, *n* = 52, *P*< 0.001)(S2 Fig). Females with S-type X-island mated with recombinant males with matching S-type X-island 81% of the times (Chi-square = 19.2, *n* = 47, *P*< 0.001)(S2 Fig).

However, the mating assays were further improved in terms of percentage of assortative mating by using exactly 5-day-old virgin female and male mosquitoes (see results). The RbMM and RbSS strains and the parental Mopti and Kisumu strains were then characterized in assortative mating experiments designed to test both female and male mate preferences. For recombinants strains, recombinant females from the RbMM or RbSS strains were given a choice of virgin males with X-islands matching their own type or not. Male choice experiments were the exact reciprocal of female choice experiments. Experiments were repeated twice and the female and the male choice for both strains (4 mating combinations) tested each time with mosquitoes of the exact same cohort and age from both strains so as to avoid potential confounding factors due to variation in body size or phenotypic quality. The mating preferences of females and males from the parental Mopti and Kisumu strains were tested using the exact same methodology and experimental design with females and males choosing between individuals of the opposite sex from their own strain or not.

### Dissection of mated females and genetic analysis of sperm

Females were dissected in order to determine their mating status and to determine the rDNA type of the male they mated with. Their spermatheca was isolated, broken open, and the sperm bundle transferred to a 1.5ml centrifuge tube as described in previous studies [18,20]. DNA extractions were done using the ChargeSwitch gDNA Micro Tissue Kit (Life Technologies, USA) following the manufacturer's instructions. The sperm DNA was genotyped using the PCR-RFLP diagnostic as described elsewhere [52].

### Genome sequencing of recombinant and parental strains

Archived DNA from 13 Mopti, 24 RbMM and 23 RbSS individuals was amplified by multiple-displacement amplification using the Illustra GenomiPhiV2 DNA Amplification kit (GE Healthcare Bio-sciences, Piscataway, NJ), purified using a MinElute Reaction Cleanup Kit (Qiagen, Hilden, Germany) and DNA pools were sent to the Liverpool Centre for Genomic Research (CGR) for sequencing. DNA libraries were prepared according to the Illumina TruSeq DNA protocol (Illumina, San Diego, CA), multiplexed and sequenced on two lanes of an Illumina HiSeq 2000 sequencer.

Base-calling of indexed reads was performed with the program CASAVA 1.8.2 (Illumina). The reads were trimmed using the software Cutadapt 1.2.1 [55] and Sickle 1.200 [56] and mapped to the *An*. *gambiae* (PEST) reference sequence (assembly AgamP3) using Bowtie 2.1.0 [57]. Alignments were filtered to remove low mapping quality reads and redundant duplicate reads were filtered out using the Picard MarkDuplicates Tool 1.85 (http://picard.sourceforge.net). Mapped reads were locally re‐aligned around indels using the Genome Analysis Tool Kit (GATK) version 2.1.13 [58,59]. The mean coverage depth after local re‐alignment and duplicate removal of reads was equal to 25.3x for RbSS, 30.9x for RbMM and 34.1x for the Mopti parental strain. Variant detection was performed using the GATK 'UnifiedGenotyper' package [58,59] with an expected SNP heterozygosity of 0.01. An expected ploidy of 20 was used (i.e. allele frequencies calculated in increments of 5%) in order to best balance accurate sample representation and computational efficiency. Variants were further filtered using the GATK 'VariantFiltration' package [58,59]. This resulted in the characterization of ~6 million SNPs (~4.8 million passing all filters) and 900,000 indels in each of the sequenced strains. All variants were annotated using snpEff 3.1 [60]. Visual alignment inspections were performed using the Integrative Genomics Viewer (IGV)[61].

Estimates of genetic differentiation *F*_*ST*_ between two populations *a* and *b* were calculated based on SNPs satisfying GATK's most stringent 'pass' criteria and using the formula:

*F*_*ST*_ = 1- *H*_*s*_/*H*_*t*_ where *Hs* is the mean heterozygosity across populations *a* and *b* and *H*_*t*_, the total heterozygosity across all populations [62].

*H*_*s*_ = 1- Σ*pi* ^2^ where *pi* are the mean frequencies of the major and minor alleles calculated from the *a* or *b* populations and:

*Ht* = 1- Σ*pi* ^2^ with *pi* being SNP frequencies calculated across all five populations.

Pair-wise *F*_*ST*_ estimates of genetic differentiation were used for generating scans of genetic differentiation across chromosomes with the software JMP 10 (SAS Institute, Inc). In order to best outline the genomic region(s) introgressed from *An*. *gambiae s*.*s*. into *An*. *coluzzii* in the RbSS and RbMM strains, the 'spline' function was fitted over every high-confidence SNPs with *δ* = 2.72^16^.

Separate data analyses identified all unique protein-coding differences between the RbSS, RbMM and Mopti strains. These differences were checked by visual inspection and comparison of their genomes using the software IGV.

### Deep targeted sequencing of field sympatric populations

*Anopheles gambiae* s.l. larvae were collected in Akoti-Chirano (Lat. 6° 6’ 17.08” N; Long. 2° 19’ 0.45” W) in the Bibiani-Anhwiaso-Bekwai district of the Western Region of Ghana, West Africa. Populations of *An*. *coluzzii* and *An*. *gambiae* s.s. co-occur in this deciduous forested area and are both of the 'Forest chromosomal form' characterized by standard karyotypic arrangements (no paracentric inversions) [63]. Larvae were reared to adulthood at the Department of Animal Biology and Conservation Science, University of Ghana, Legon, West Africa, stored in ethanol, and shipped to Keele University. The samples were then individually characterized as *An*. *coluzzii* (M molecular form) and *An*. *gambiae* s.s. (S molecular form) as described above. The DNA from 30 individuals of each sibling species was pooled and purified using a MinElute Reaction Cleanup Kit (Qiagen, Hilden, Germany) and DNA pools were sent to the Liverpool Centre for Genomic Research (CGR) for sequence capture and sequencing.

SureSelect RNA oligomer baits (Agilent, Santa Clara, CA) were designed to cover coding regions from position 17Mb to the centromere of the X-chromosome based on the (PEST) reference sequence (assembly AgamP3). Prior to the amplification of pre-capture libraries, DNA fragments larger than ∼700bp were removed from DNA pools using Agencourt AMPure XP beads (Beckman Coulter, Brea, CA). Following amplification and adapter-ligation, 750ng of pre‐capture libraries were hybridised to 2μl of RNA oligomer baits for ∼24 hours at 65°C. Captured libraries were amplified, indexed, pooled and sequenced on 1 lane of an Illumina HiSeq 2000. All other procedures were as described above. Within the targeted pericentric region of the X chromosome, the mean coverage depth after local re‐alignment and duplicate removal of low mapping quality and redundant reads was 200x for *An*. *gambiae* s.s., leading to the identification of 26,974 SNPS and 2,593 indels. In *An*. *coluzzii*, coverage depth was 255x and 18,772 SNPS and 1,738 indels were identified.

### Statistical analyses

All statistical analyses were performed using the software JMP 10 (SAS Institute, Inc). Pearson Chi-square tests of randomness and goodness of fit (likelihood ratios) were used for detecting deviations from random mating in assortative mating experiments and to compare observed M and S X-chromosome frequencies to expected Mendelian and Hardy-Weinberg Equilibrium (HWE) proportions at different generations of the genetic crosses.

## Supporting Information

S1 Fig Frequency of MS and MM genotypes at the diagnostic X-chromosome island of speciation locus. Pooled larval genotyping was performed on the female progeny for backcross generations 1–4 and the 1^st^ generation of cross within introgressed strains (generation 5) of the X-island loci in recombinant strains. Significant deviations from the expected Mendelian ratios (1:1 MS/MM for backcrosses and 3:1 for 1^st^ fixation generation) are indicated (Goodness of fit test: *** = *P*<0.001, ns = non-significant).
(TIF)

S2 Fig Overall percentage assortative mating in females from recombinant strains carrying M or S type X-chromosome islands in preliminary mating experiments. Thirty 2–5 day-old virgin females where presented with a mixture of 2-5-day-old recombinant males matching and non-matching their own X-island molecular types in standardized overnight mating assay.
(TIF)

S1 Table Number of females and males mating assortatively in reciprocal behavioural assays among the Mopti and Kisumu parental strains. Females (X-island genotypes MM or SS) were given a choice between males from their own strain and molecular type or not (top half of table, see methods). The reciprocal experiments were also conducted with males (X-island genotypes M or S) choosing females (bottom half of table). The number of replicates, mating combinations, numbers and percentages (in brackets) of mating, and level of significance (Chi-square Likelihood-ratios) are indicated.
(DOCX)

S2 Table Number of females mating assortatively and disassortatively in preliminary behavioural assays among recombinants strains. Recombinant females (X-island genotypes MM or SS) were given a choice between recombinant males with X-chromosome speciation island matching their own or not (see methods). The number of replicates, mating combinations, numbers and percentages (in brackets) of mating, and level of significance (Chi-square Likelihood-ratios) are indicated.
(DOCX)
