# Supplementary figures and images for: Experimental Swap of Anopheles gambiae's Assortative Mating Preferences Demonstrates Key Role of X-Chromosome Divergence Island in Incipient Sympatric Speciation
Source: PLoS Genet. 2015 Apr 16;11(4):e1005141. doi: 10.1371/journal.pgen.1005141 (PMC4400153; doi:10.1371/journal.pgen.1005141)

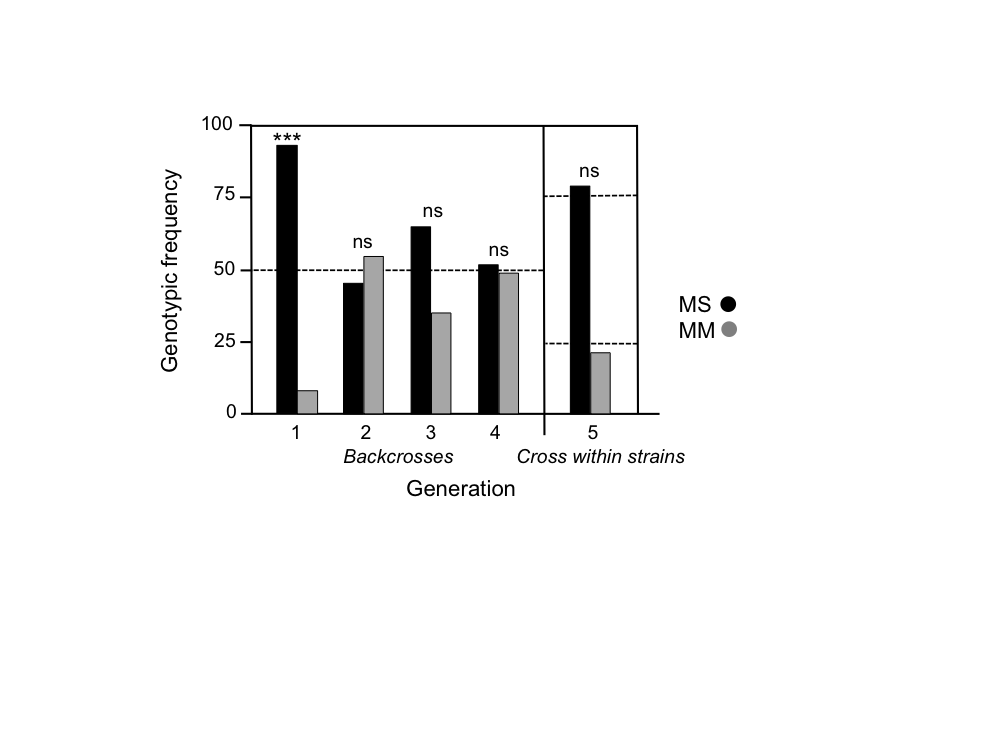

Supplement: S1 Fig — Pooled larval genotyping was performed on the female progeny for backcross generations 1–4 and the 1st generation of cross within introgressed strains (generation 5) of the X-island loci in recombinant strains. Significant deviations from the expected Mendelian ratios (1:1 MS/MM for backcrosses and 3:1 for 1st fixation generation) are indicated (Goodness of fit test: *** = P<0.001, ns = non-significant). (TIF) [file pgen.1005141.s001.tif]

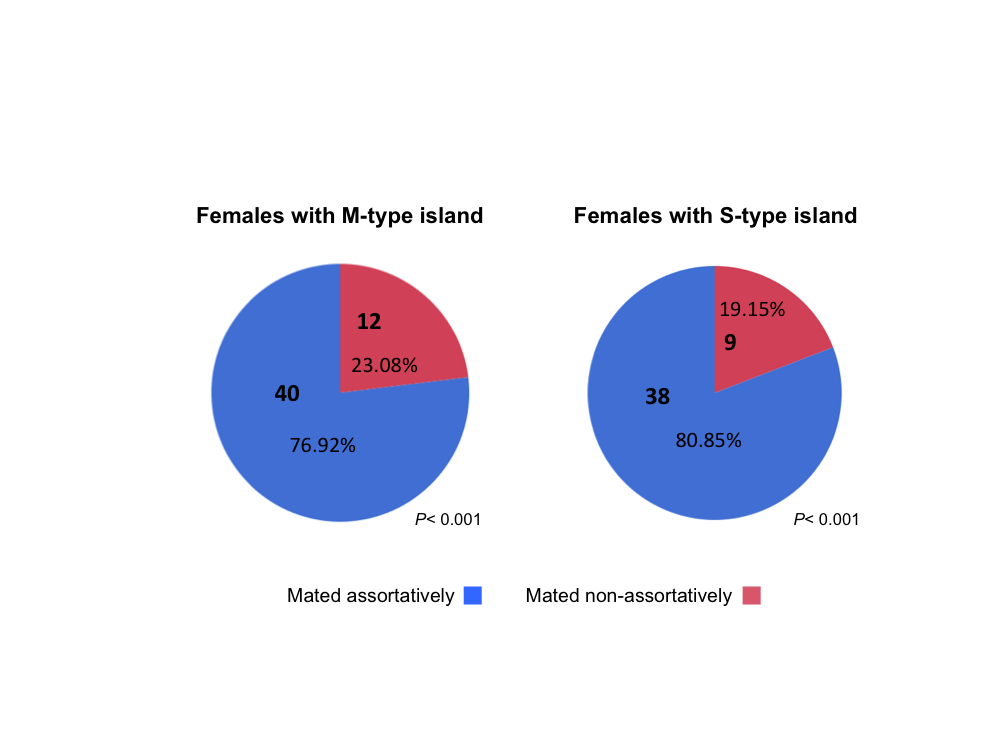

Supplement: S2 Fig — Thirty 2–5 day-old virgin females where presented with a mixture of 2-5-day-old recombinant males matching and non-matching their own X-island molecular types in standardized overnight mating assay. (TIF) [file pgen.1005141.s002.tif]
