# Supplementary material for: Experimental Swap of Anopheles gambiae's Assortative Mating Preferences Demonstrates Key Role of X-Chromosome Divergence Island in Incipient Sympatric Speciation
Source: PLoS Genet. 2015 Apr 16;11(4):e1005141. doi: 10.1371/journal.pgen.1005141 (PMC4400153; doi:10.1371/journal.pgen.1005141)
Supplement: S1 Table — Females (X-island genotypes MM or SS) were given a choice between males from their own strain and molecular type or not (top half of table, see methods). The reciprocal experiments were also conducted with males (X-island genotypes M or S) choosing females (bottom half of table). The number of replicates, mating combinations, numbers and percentages (in brackets) of mating, and level of significance (Chi-square Likelihood-ratios) are indicated. (DOCX) [file pgen.1005141.s003.docx]

| **Replicate** | **Mating combination** | | | | **Mating Type (%)** | | **Chi-square** | ***P*-value** |
| --- | --- | --- | --- | --- | --- | --- | --- | --- |
|  | **Females^†^** | | **Males^†^** | | **Assortative** | **Disassortative** |  |  |
| 1 | MM | | M | S | 18 | 5 | 7.8 | 0.005 |
| 2 | MM | | M | S | 19 | 2 | 15.9 | < 0.001 |
| 3 | MM | | M | S | 20 | 1 | 27.1 | < 0.001 |
| *All* |  | |  |  | *57 (87.69)* | *8 (12.30)* | *41.6* | *< 0.001* |
| 1 | SS | | M | S | 20 | 5 | 9.6 | 0.002 |
| 2 | SS | | M | S | 28 | 0 | - | < 0.001 |
| 3 | SS | | M | S | 14 | 6 | 3.3 | 0.070 |
| *All* |  | |  |  | *62 (84.93)* | *11 (15.07)* | *39.3* | *< 0.001* |
|  |  | |  |  |  |  |  |  |
| **Replicate** | **Mating combination** | | | | **Mating Type (%)** | | **Chi-square** | ***P*-value** |
|  | **Males^†^** | **Females^†^** | | | **Assortative** | **Disassortative** |  |  |
| 1 | M | MM | | SS | 15 | 21 | 1.0 | 0.316 |
| 2 | M | MM | | SS | 10 | 15 | 1.0 | 0.316 |
| 3 | M | MM | | SS | 12 | 14 | 0.2 | 0.695 |
| *All* |  |  | |  | *37 (42.53)* | *50 (57.47)* | *1.9* | *0.163* |
| 1 | S | MM | | SS | 22 | 9 | 5.6 | 0.018 |
| 2 | S | MM | | SS | 11 | 13 | 0.2 | 0.683 |
| 3 | S | MM | | SS | 15 | 10 | 1.0 | 0.316 |
| *All* |  |  | |  | *48 (60.00)* | *32 (40.00)* | *3.2* | *0.073* |

**^†^** For each mating combinations, 3 replicates were conducted using 5-day-old mosquitoes reared from independent mosquito cohorts.
