# Supplementary material for: Experimental Swap of Anopheles gambiae's Assortative Mating Preferences Demonstrates Key Role of X-Chromosome Divergence Island in Incipient Sympatric Speciation
Source: PLoS Genet. 2015 Apr 16;11(4):e1005141. doi: 10.1371/journal.pgen.1005141 (PMC4400153; doi:10.1371/journal.pgen.1005141)
Supplement: S2 Table — Recombinant females (X-island genotypes MM or SS) were given a choice between recombinant males with X-chromosome speciation island matching their own or not (see methods). The number of replicates, mating combinations, numbers and percentages (in brackets) of mating, and level of significance (Chi-square Likelihood-ratios) are indicated. (DOCX) [file pgen.1005141.s004.docx]

| **Replicate** | **Mating combination** | | | **Mating Type (%)** | | **Chi-square** | ***P*-value** |
| --- | --- | --- | --- | --- | --- | --- | --- |
|  | **Females^†^** | **Males^†^** | | **Assortative** | **Disassortative** |  |  |
| 1 | MM | M | S | 12 | 2 | 7.9 | 0.005 |
| 2 | MM | M | S | 7 | 4 | 0.8 | 0.362 |
| 3 | MM | M | S | 10 | 3 | 4.0 | 0.046 |
| 4 | MM | M | S | 11 | 3 | 4.9 | 0.028 |
| *All* |  |  |  | *40 (76.92)* | *12 (23.08)* | *15.9* | *< 0.001* |
| 1 | SS | M | S | 9 | 2 | 4.8 | 0.028 |
| 2 | SS | M | S | 10 | 6 | 1.0 | 0.314 |
| 3 | SS | M | S | 13 | 1 | 12.2 | 0.001 |
| 4 | SS | M | S | 6 | 0 | 8.3 | 0.004 |
| *All* |  |  |  | *38 (80.85)* | *9 (19.15)* | *19.2* | *< 0.001* |

**^†^** For each mating combinations, 4 replicates were conducted using mosquitoes 2-5-day-old reared from independent mosquito cohorts.
